# Supplementary figures and images for: Immigrant birds learn from socially observed differences in payoffs when their environment changes
Source: PLoS Biol. 2024 Nov 14;22(11):e3002699. doi: 10.1371/journal.pbio.3002699 (PMC11563421; doi:10.1371/journal.pbio.3002699)

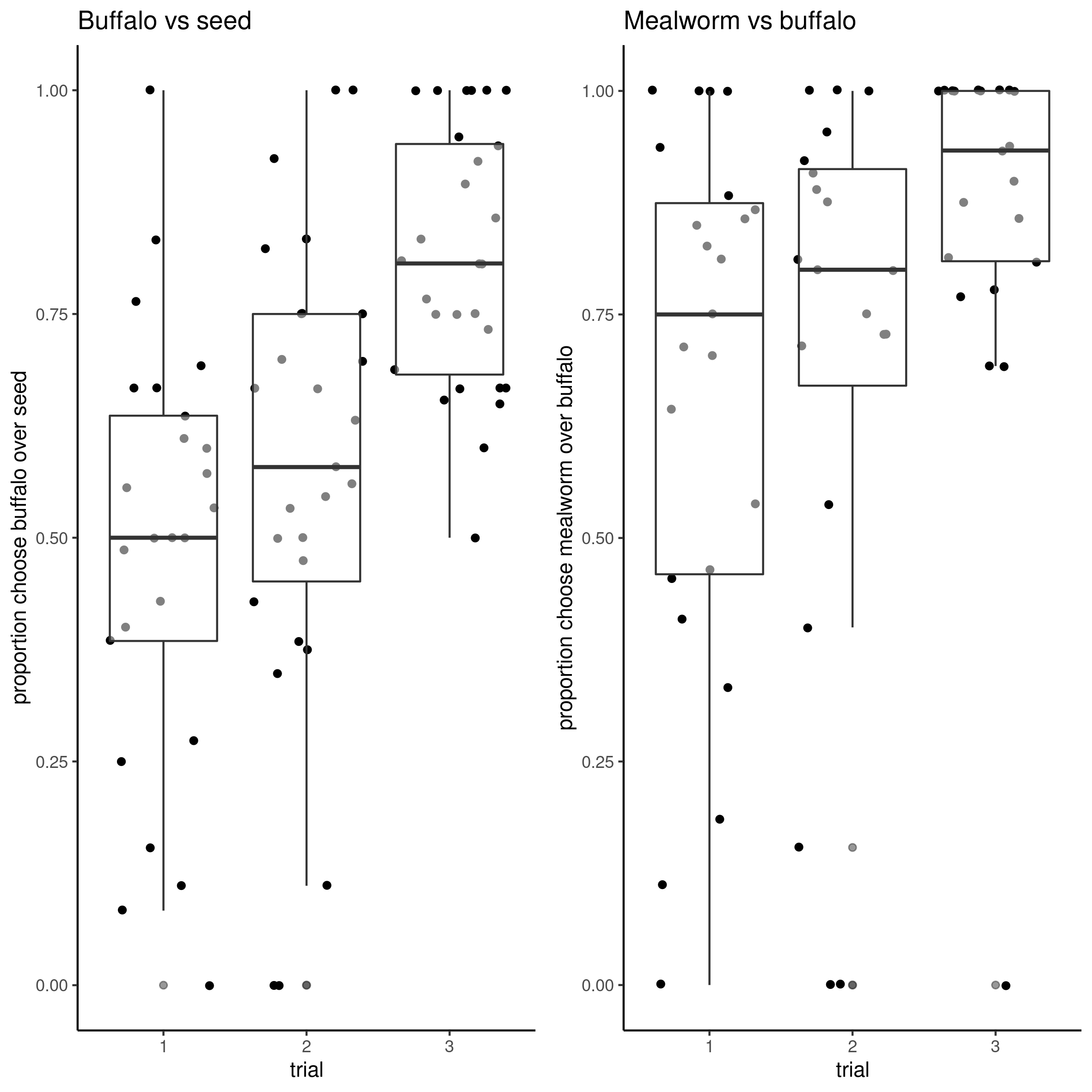

Supplement: S1 Fig — Proportion of choices (y-axis) by trial (x-axis), where each dot represents an individual bird. Birds preferred buffalo worms to seed, and mealworms to buffalo mealworms, and these preferences strengthened over time. The data underlying this figure can be found in our data and code repository (https://doi.org/10.17617/3.FXC12W). (PNG) [file pbio.3002699.s001.png]

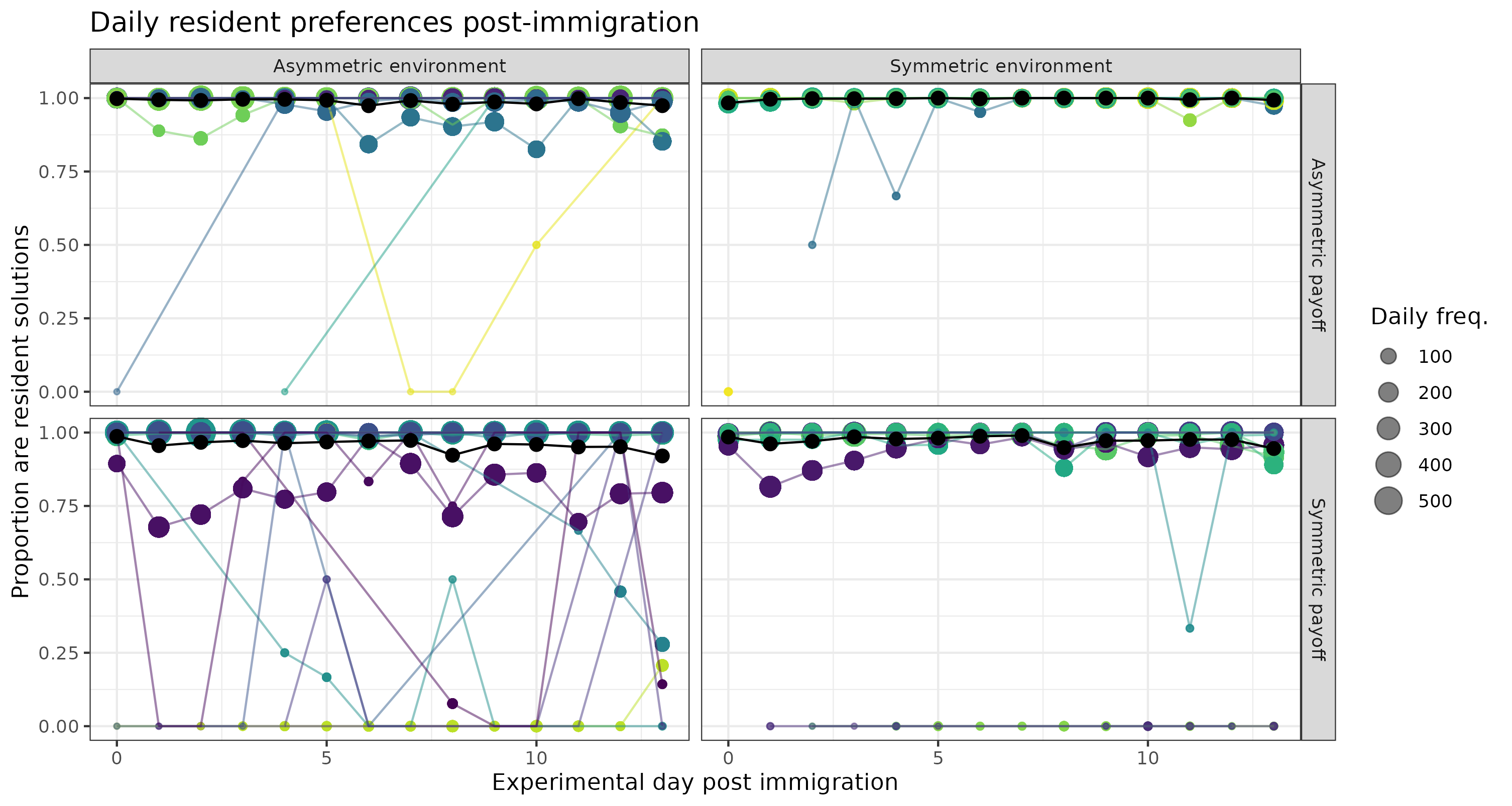

Supplement: S2 Fig — Daily preferences of residents after immigration. Proportion of immigrant solutions which were resident solutions (y-axis) over experimental day (x-axis). Colored lines are individual immigrants, with the daily solving frequency indicated (size). A handful of residents in the Ps conditions adopted the immigrant preference; however, they had either learned to use the puzzle after immigration, or had very low solving rates. The data underlying this figure can be found in our data and code repository (https://doi.org/10.17617/3.FXC12W). (PNG) [file pbio.3002699.s002.png]

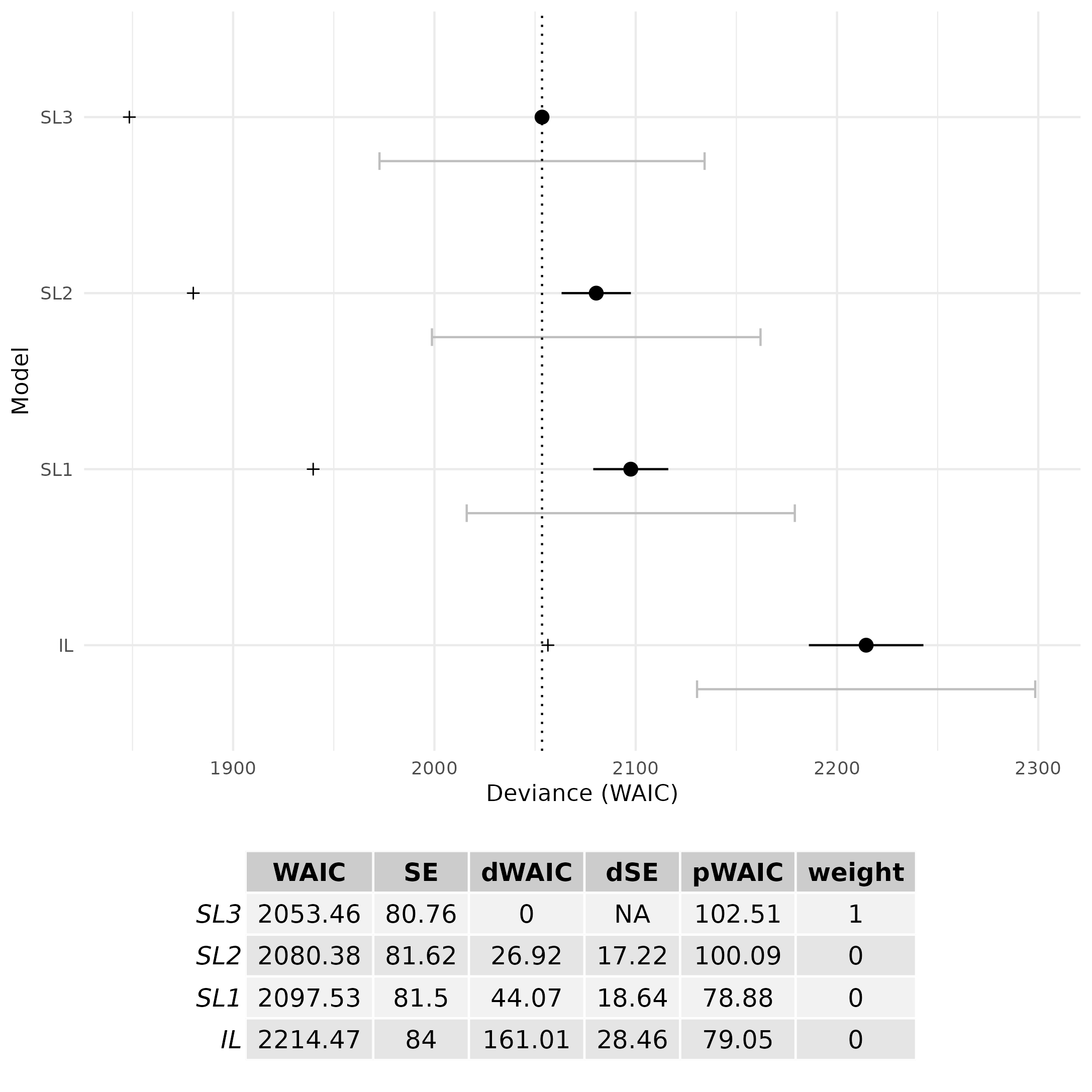

Supplement: S3 Fig — WAIC comparison of dynamic learning models. Black dots indicate out-of-sample deviance, “+” symbols indicate in-sample deviance. Gray bars indicate standard error, and black bars are the difference in standard errors. Vertical dotted line is the WAIC of the highest ranked model. Models with social learning components were better fit than individual learning alone, and the highest ranked model estimated payoff-biased social learning, a new associate bias, and a sloped change to the sensitivity to social information. The data underlying this figure can be found in our data and code repository (https://doi.org/10.17617/3.FXC12W). (PNG) [file pbio.3002699.s003.png]

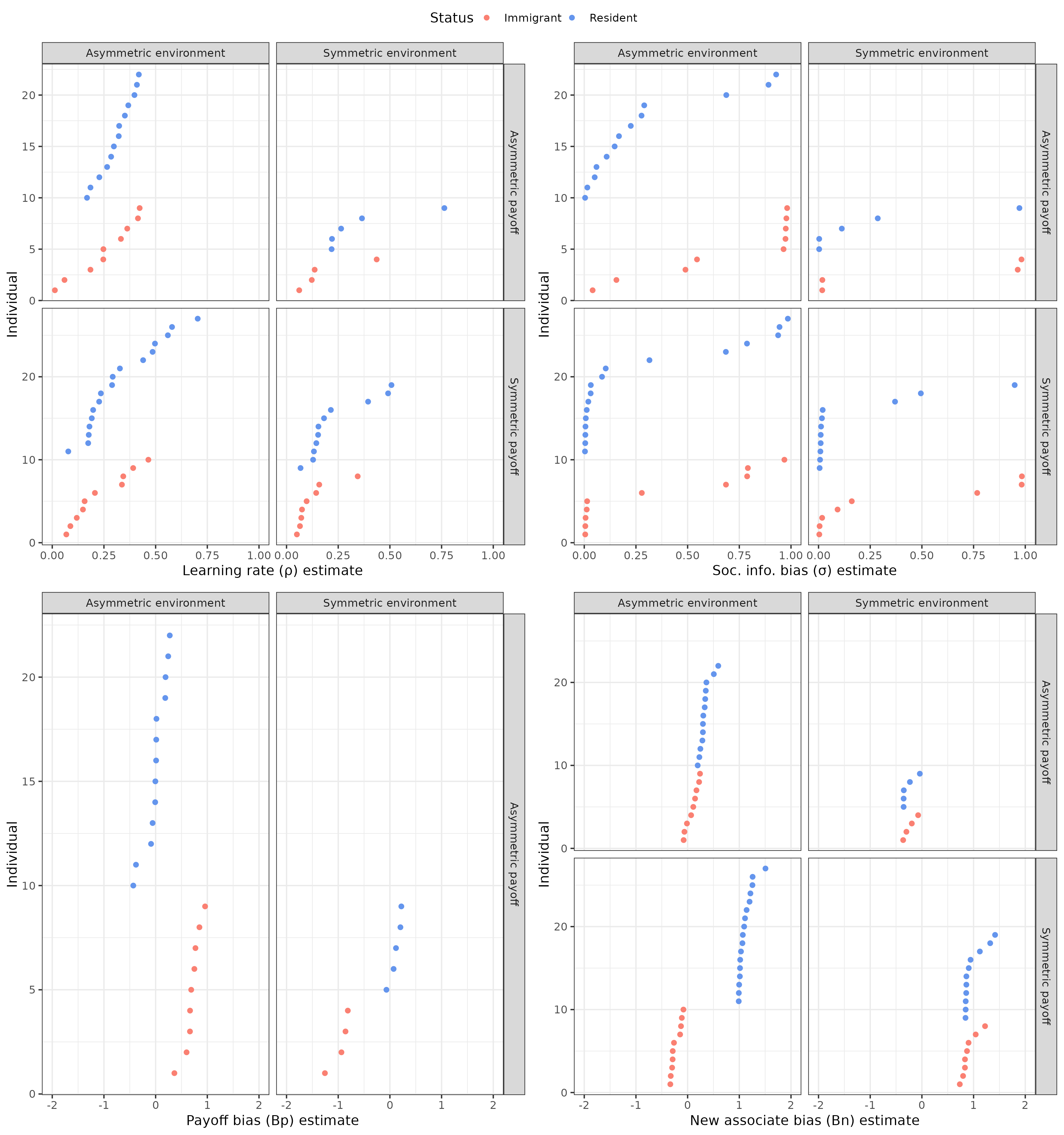

Supplement: S4 Fig — Point estimates of individual birds from Bayesian learning model SL3 (immigrant status as color). Point estimates shown for learning rate (ρ), social information bias (σ), payoff bias (βp), new associate bias (βn). The data underlying this figure can be found in our data and code repository (https://doi.org/10.17617/3.FXC12W). (PNG) [file pbio.3002699.s004.png]

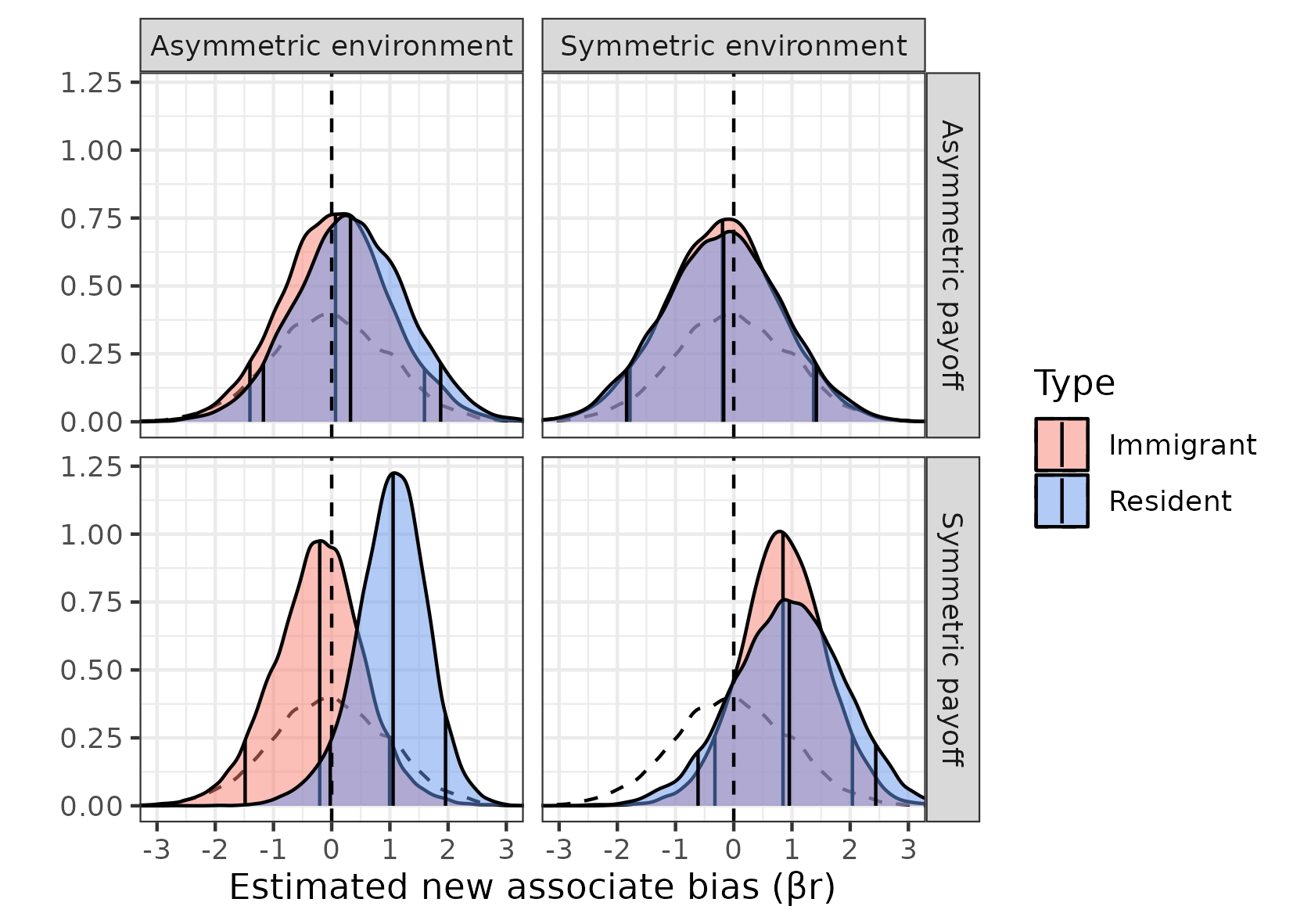

Supplement: S5 Fig — Posterior distributions of an estimated new associate bias for residents and immigrants. Estimates above zero indicate that birds were disproportionately influenced by observations of solves by new associates. Estimates below zero indicate that individuals disproportionately ignored observations of solves by new associates. The data underlying this figure can be found in our data and code repository (https://doi.org/10.17617/3.FXC12W). (PNG) [file pbio.3002699.s005.png]

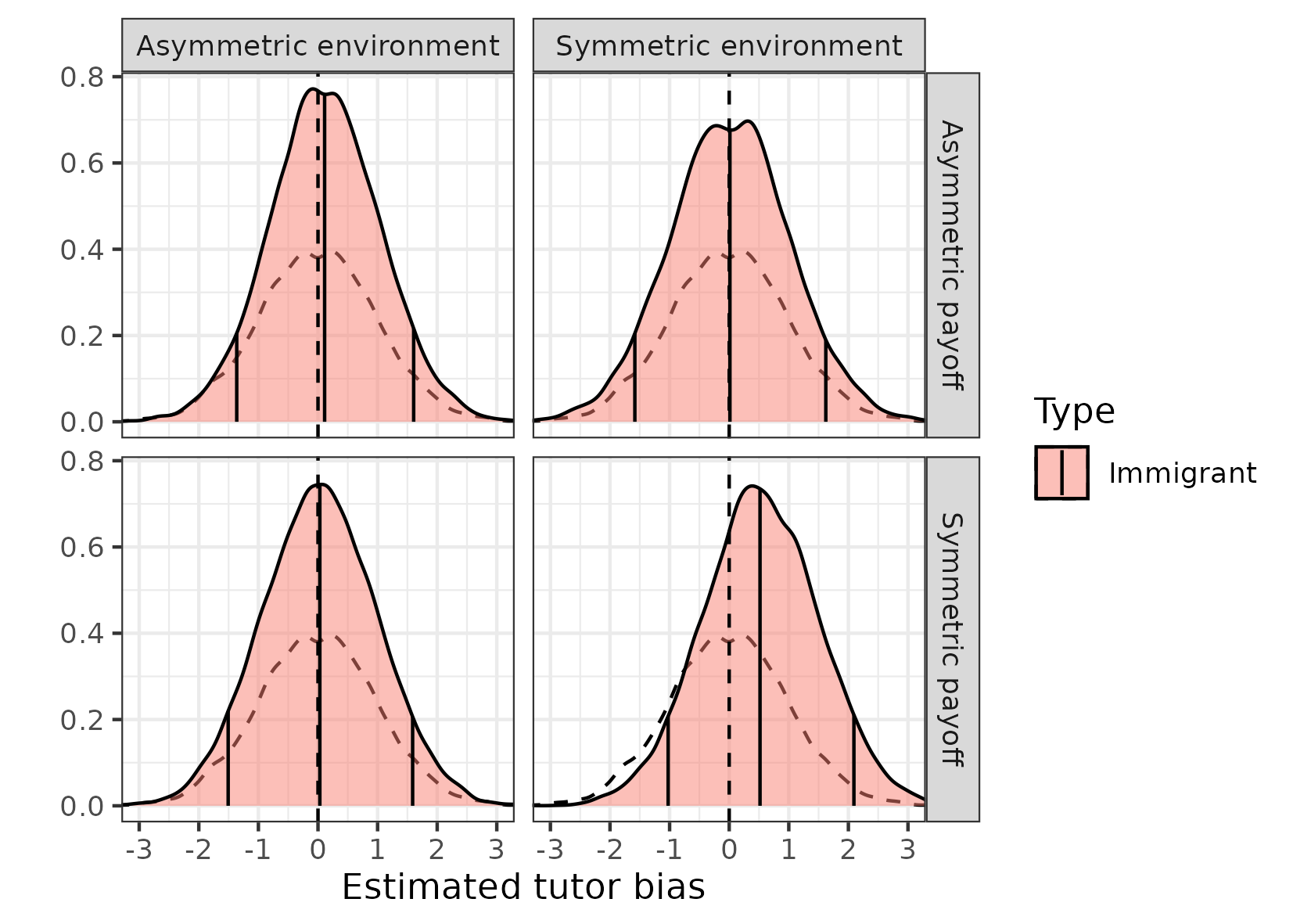

Supplement: S6 Fig — We fit an additional model, similar to SL3, except that rather than information about whether a solution was produced by a new associate, we included information about whether a solution was produced by a tutor. This was done to determine whether immigrants were disproportionately influenced by the productions of tutors in their new populations. We found no evidence to support this in any condition. The data underlying this figure can be found in our data and code repository (https://doi.org/10.17617/3.FXC12W). (PNG) [file pbio.3002699.s006.png]
