# Supplementary material for: Immigrant birds learn from socially observed differences in payoffs when their environment changes
Source: PLoS Biol. 2024 Nov 14;22(11):e3002699. doi: 10.1371/journal.pbio.3002699 (PMC11563421; doi:10.1371/journal.pbio.3002699)
Supplement: S1 Text — Table A. Relative value of food items. Table B. Non-tutor birds preferred the seeded solution. Table C. How did immigrant preferences change after the immigration event? Table D. Summary of model estimates, individual learning only (IL). Table E. Summary of model estimates, SL1. Table F. Summary of model estimates, SL2. Table G. Summary of model estimates, SL3. (PDF) [file pbio.3002699.s007.pdf]

Supplementary text for manuscript "Immigrant birds learn from socially observed differences in payoffs when their environment changes"

Authors: Michael Chimento, Gustavo Alarcón-Nieto, Lucy M. Aplin

Tables

- **Table A**: Relative value of food items.
- **Table B**: Non-tutor birds preferred the seeded solution.
- **Table C**: How did immigrant preferences change after the immigration event?
- **Table D**: Summary of model estimates, individual learning only (IL)
- **Table E**: Summary of model estimates, SL1
- **Table F**: Summary of model estimates, SL2
- **Table G**: Summary of model estimates, SL3

Table A: Relative value of food items. Value estimates from Bayesian inverse reinforcement learning model. Reported values are mean (89% HDPI). The data underlying this table can be found in our data and code repository https://doi.org/10.17617/3.FXC12W.

parameter	value	n. eff.	Rhat
reward_seeds	0.35 (0.02,1.02)	1275.45	1.00
reward_buffalo	1.72 (1.33,2.39)	1294.75	1.00
reward_mealworms	3.66 (3.12,4.4)	1444.63	1.00

Table B: Non-tutor birds preferred the seeded solution. Linear mixed model where the response variable was the proportion of solutions by non-tutors during the diffusion period that were of the seeded side. There were no significant differences between age, sex, immigrants, residents or conditions. Year was included as a random intercept. The data underlying this table can be found in our data and code repository `https://doi.org/10.17617/3.FXC12W`.

	Dependent variable:
	Prop. seeded soln.
Age (adult)	−0.056 (0.034)
	t = −1.628
	p = 0.104
Sex (male)	0.054 (0.033)
	t = 1.614
	p = 0.107
Resident	0.048 (0.063)
	t = 0.764
	p = 0.446
Payoff condition (symmetric)	−0.070 (0.062)
	t = −1.120
	p = 0.263
Environment condition (symmetric)	0.014 (0.070)
	t = 0.195
	p = 0.846
Resident:Payoff condition	−0.070 (0.080)
	t = −0.866
	p = 0.387
Resident:Environment condition	−0.015 (0.102)
	t = −0.150
	p = 0.881
Payoff condition:Environment condition	0.052 (0.093)
	t = 0.563
	p = 0.574
Resident:Payoff condition:Environment condition	0.021 (0.130)
	t = 0.163
	p = 0.871
Intercept	0.995 (0.067)
	t = 14.842
	p = 0.000***
Observations	71
Log Likelihood	27.199
Akaike Inf. Crit.	−30.397
Bayesian Inf. Crit.	−3.245
Note:	*p<0.1; **p<0.05; ***p<0.01

Table C: How did immigrant preferences change after the immigration event? Logistic GLMM where the probability of producing the resident solve was predicted by normalized experience solving the puzzle pre-immigration (0 representing the mean number of solutions produced of the resident side of an immigrants original population), and the interaction of days since immigration (starting at 0), payoff condition and environmental condition. The intercept can be interpreted as the probability of producing the resident solution of a bird with average experience of solving on the day immediately following immigration. The data underlying this table can be found in our data and code repository`https://doi.org/10.17617/3.FXC12W`.

	Dependent variable:
	Produce resident soln.
Normalized experience	−1.036 (0.674)
	t = −1.538
	p = 0.125
Day since immigration	0.223 (0.008)
	t = 26.788
	p = 0.000***
Environment condition (symmetric)	−2.156 (2.367)
	t = −0.911
	p = 0.363
Payoff condition (symmetric)	−6.550 (1.721)
	t = −3.805
	p = 0.0002***
Day:Environment condition	0.431 (0.023)
	t = 18.413
	p = 0.000***
Day:Payoff condition	−0.032 (0.013)
	t = −2.427
	p = 0.016**
Payoff condition:Environment condition	3.956 (2.948)
	t = 1.342
	p = 0.180
Day:Environment condition:Payoff condition	−0.765 (0.034)
	t = −22.450
	p = 0.000***
Intercept	2.153 (1.232)
	t = 1.747
	p = 0.081*
Observations	40,363
Log Likelihood	−8,000.502
Akaike Inf. Crit.	16,023.000
Bayesian Inf. Crit.	16,117.670
Note:	*p<0.1; **p<0.05; ***p<0.01

Table D: Summary of model estimates, individual learning only (IL). Table contains mean values (89% HDPI), number of effective samples, and Gelman's Rhat. Parameter estimates and SD of varying effects across individuals are reported. The data underlying this table can be found in our data and code repository`https://doi.org/10.17617/3.FXC12W`.

condition	value	n. eff.	Rhat
**Recent information bias ρ**			
E_a, P_a res.	0.32 (0.14,0.58)	7828.97	1.00
E_a, P_a immi.	0.29 (0.14,0.52)	5312.50	1.00
E_a, P_s res.	0.28 (0.14,0.5)	7424.18	1.00
E_a, P_s immi.	0.22 (0.09,0.45)	6048.09	1.00
E_s, P_a res.	0.33 (0.12,0.64)	10894.80	1.00
E_s, P_a immi.	0.29 (0.11,0.6)	6167.35	1.00
E_s, P_s res.	0.27 (0.12,0.5)	8152.20	1.00
E_s, P_s immi.	0.16 (0.06,0.38)	7480.34	1.00
**Behavioral conservatism α**			
	1.29 (1.05,1.59)	3693.88	1.00
**SD individals**			
rho (logit scale)	2.13 (1.49,2.93)	3772.24	1.00
alpha (log scale)	0.97 (0.76,1.23)	4401.25	1.00

Table E: Summary of model estimates, SL1. Table contains mean values (89% HDPI), number of effective samples, and Gelman's Rhat. Parameter estimates and SD of varying effects across individuals are reported. The data underlying this table can be found in our data and code repository`https://doi.org/10.17617/3.FXC12W`.

condition	value	n. eff.	Rhat
**Recent information bias ρ**			
E_a, P_a res.	0.43 (0.19,0.71)	9693.42	1.00
E_a, P_a immi.	0.3 (0.13,0.58)	4360.72	1.00
E_a, P_s res.	0.47 (0.24,0.71)	5511.47	1.00
E_a, P_s immi.	0.33 (0.14,0.6)	4454.35	1.00
E_s, P_a res.	0.46 (0.19,0.75)	11384.45	1.00
E_s, P_a immi.	0.32 (0.12,0.63)	5468.66	1.00
E_s, P_s res.	0.38 (0.17,0.65)	5507.65	1.00
E_s, P_s immi.	0.21 (0.07,0.5)	4216.93	1.00
**Social information bias σ**			
E_a, P_a res.	0.24 (0.07,0.58)	14778.65	1.00
E_a, P_a immi.	0.27 (0.08,0.61)	15717.76	1.00
E_a, P_s res.	0.13 (0.03,0.38)	11849.47	1.00
E_a, P_s immi.	0.15 (0.04,0.44)	14627.14	1.00
E_s, P_a res.	0.24 (0.06,0.6)	16923.20	1.00
E_s, P_a immi.	0.28 (0.08,0.64)	16501.87	1.00
E_s, P_s res.	0.14 (0.03,0.42)	14985.13	1.00
E_s, P_s immi.	0.23 (0.06,0.58)	15753.78	1.00
**Behavioral conservatism α**			
	1.45 (1.08,1.96)	3477.45	1.00
**SD individals**			
rho (logit scale)	2.49 (1.67,3.47)	3225.22	1.00
sigma (logit scale)	7.32 (5.19,10.01)	6499.56	1.00
alpha (log scale)	0.97 (0.7,1.3)	4030.65	1.00

Table F: **Summary of model estimates, SL2.** Table contains mean values (89% HDPI), number of effective samples, and Gelman's Rhat. Parameter estimates and SD of varying effects across individuals are reported. The data underlying this table can be found in our data and code repository`https://doi.org/10.17617/3.FXC12W`.

condition	value	n. eff.	Rhat
**Recent information bias ρ**			
E_a, P_a res.	0.4 (0.17,0.69)	12083.94	1.00
E_a, P_a immi.	0.26 (0.1,0.54)	7302.48	1.00
E_a, P_s res.	0.45 (0.23,0.71)	8683.09	1.00
E_a, P_s immi.	0.32 (0.13,0.59)	6662.80	1.00
E_s, P_a res.	0.46 (0.18,0.77)	18116.73	1.00
E_s, P_a immi.	0.33 (0.12,0.63)	8183.72	1.00
E_s, P_s res.	0.35 (0.14,0.63)	9591.48	1.00
E_s, P_s immi.	0.22 (0.07,0.51)	7797.58	1.00
**Social information bias σ**			
E_a, P_a res.	0.25 (0.07,0.6)	18884.55	1.00
E_a, P_a immi.	0.3 (0.09,0.65)	20337.34	1.00
E_a, P_s res.	0.12 (0.03,0.37)	16612.20	1.00
E_a, P_s immi.	0.16 (0.04,0.47)	19557.11	1.00
E_s, P_a res.	0.25 (0.07,0.61)	25372.26	1.00
E_s, P_a immi.	0.28 (0.08,0.64)	23219.85	1.00
E_s, P_s res.	0.16 (0.04,0.48)	11654.37	1.00
E_s, P_s immi.	0.23 (0.06,0.58)	22053.23	1.00
**Payoff bias β_p**			
E_a, P_a res.	0.22 (-1.14,1.49)	10109.01	1.00
E_a, P_a immi.	1 (-0.11,2.17)	14580.06	1.00
E_a, P_s res.	0.14 (-1.05,1.2)	9189.89	1.00
E_a, P_s immi.	-0.04 (-1.46,1.38)	25623.98	1.00
E_s, P_a res.	0.24 (-1.31,1.71)	19833.64	1.00
E_s, P_a immi.	-0.49 (-1.9,0.92)	20427.56	1.00
E_s, P_s res.	-0.25 (-1.54,1)	16405.30	1.00
E_s, P_s immi.	-0.36 (-1.67,0.94)	18126.53	1.00
**New associate bias β_n**			
E_a, P_a res.	0.3 (-1.19,1.8)	23100.79	1.00
E_a, P_a immi.	0.43 (-1.04,1.91)	24623.23	1.00
E_a, P_s res.	0.65 (-0.69,1.81)	5928.67	1.00
E_a, P_s immi.	-0.71 (-2.23,0.85)	15223.34	1.00
E_s, P_a res.	-0.09 (-1.64,1.53)	27264.13	1.00
E_s, P_a immi.	-0.13 (-1.69,1.45)	26157.39	1.00
E_s, P_s res.	0.41 (-1.36,2.21)	2827.70	1.00
E_s, P_s immi.	0.24 (-1.19,1.64)	9807.23	1.00
**Behavioral conservatism α**			
	1.46 (1.08,2)	4551.06	1.00
**SD individals**			
rho (logit scale)	2.43 (1.59,3.47)	4635.11	1.00
sigma (logit scale)	7.01 (4.97,9.66)	4996.06	1.00
β_p	1.04 (0.74,1.43)	3172.60	1.00
β_n	1.08 (0.1,2.39)	4230.11	1.00
alpha (log scale)	1.92 (0.17,4.14)	1450.48	1.00

Table G: Summary of model estimates, SL3. Table contains mean values (89% HDPI), number of effective samples, and Gelman's Rhat. Parameter estimates and SD of varying effects across individuals are reported. The data underlying this table can be found in our data and code repositoryhttps://doi.org/10.17617/3.FXC12W.

condition	value	n. eff.	Rhat
**Recent information bias ρ**			
E_a, P_a res.	0.3 (0.13,0.57)	5949.42	1.00
E_a, P_a immi.	0.2 (0.09,0.42)	3406.64	1.00
E_a, P_s res.	0.32 (0.16,0.56)	4761.78	1.00
E_a, P_s immi.	0.23 (0.1,0.46)	5039.94	1.00
E_s, P_a res.	0.42 (0.17,0.72)	10619.61	1.00
E_s, P_a immi.	0.23 (0.08,0.52)	4443.83	1.00
E_s, P_s res.	0.25 (0.11,0.49)	6560.86	1.00
E_s, P_s immi.	0.12 (0.04,0.31)	5259.67	1.00
**Social information bias σ**			
E_a, P_a res.	0.26 (0.07,0.62)	18099.07	1.00
E_a, P_a immi.	0.49 (0.18,0.81)	12818.45	1.00
E_a, P_s res.	0.18 (0.05,0.46)	14287.71	1.00
E_a, P_s immi.	0.22 (0.06,0.54)	16235.82	1.00
E_s, P_a res.	0.24 (0.07,0.6)	21053.66	1.00
E_s, P_a immi.	0.32 (0.09,0.7)	15930.05	1.00
E_s, P_s res.	0.16 (0.04,0.46)	17341.29	1.00
E_s, P_s immi.	0.29 (0.09,0.62)	14814.41	1.00
**σ slope**			
E_a, P_a res.	-0.81 (-2.14,0.43)	2905.82	1.00
E_a, P_a immi.	-1.43 (-2.55,-0.37)	5449.14	1.00
E_a, P_s res.	-1.13 (-2.26,-0.28)	5043.10	1.00
E_a, P_s immi.	-1.56 (-2.62,-0.6)	10798.47	1.00
E_s, P_a res.	-0.4 (-1.5,0.64)	7611.25	1.00
E_s, P_a immi.	-0.55 (-1.71,0.62)	2768.85	1.00
E_s, P_s res.	-1.55 (-2.8,-0.39)	9572.58	1.00
E_s, P_s immi.	-0.83 (-1.76,0.03)	5005.61	1.00
**Payoff bias β_p**			
E_a, P_a res.	0 (-1.35,1.26)	5562.69	1.00
E_a, P_a immi.	0.75 (-0.25,1.87)	12397.06	1.00
E_a, P_s res.	0.17 (-0.94,1.13)	9134.40	1.00
E_a, P_s immi.	-0.17 (-1.53,1.24)	22828.06	1.00
E_s, P_a res.	0.08 (-1.42,1.53)	13039.27	1.00
E_s, P_a immi.	-0.69 (-2.03,0.73)	9406.36	1.00
E_s, P_s res.	-0.34 (-1.73,1.02)	19267.03	1.00
E_s, P_s immi.	-0.11 (-1.32,1.09)	15553.20	1.00
**New associate bias β_n**			
E_a, P_a res.	0.34 (-1.17,1.87)	17681.93	1.00
E_a, P_a immi.	0.07 (-1.4,1.59)	20084.64	1.00
E_a, P_s res.	1.02 (-0.03,1.96)	10361.35	1.00
E_a, P_s immi.	-0.23 (-1.49,0.99)	15779.76	1.00
E_s, P_a res.	-0.19 (-1.84,1.42)	22523.01	1.00
E_s, P_a immi.	-0.2 (-1.78,1.37)	19424.41	1.00
E_s, P_s res.	0.94 (-0.61,2.44)	9078.02	1.00
E_s, P_s immi.	0.85 (-0.32,2.04)	11727.84	1.00
**Behavioral conservatism α**			
	1.76 (1.34,2.32)	2286.51	1.00
**SD individals**			
rho (logit scale)	1.67 (0.95,2.54)	2025.25	1.00
sigma (logit scale)	4.71 (2.92,6.86)	4158.91	1.00
sigma slope (logit scale)	0.89 (0.66,1.18)	4501.67	1.00
β_p	0.92 (0.08,2.14)	3328.69	1.00
β_n	0.72 (0.05,1.96)	3377.31	1.00
alpha (log scale)	1 (0.09,2.52)	1087.27	1.00
