# Supplementary material for: Immigrant birds learn from socially observed differences in payoffs when their environment changes
Source: PLoS Biol. 2024 Nov 14;22(11):e3002699. doi: 10.1371/journal.pbio.3002699 (PMC11563421; doi:10.1371/journal.pbio.3002699)
Supplement: Attachment — Submitted filename: response_to_reviewers.pdf [file pbio.3002699.s008.pdf]

Please find below our point-by-point responses (**bold**) to the Reviewers' comments (*italics*). We thank the reviewers for their time spent considering our manuscript. NB: the reference numbers in the quoted blocks do not reflect the reference numbers in the main text.

Reviewer 1

1. *This is an interesting study using an experiment on captive great tits alongside Bayesian learning models to test how social information use by immigrant individuals is influenced by changes in habitat type and reward payoffs. The experiment is very cleverly designed and the analyses are rigorous and compelling, so I have only relatively minor comments on the clarity of the manuscript.*

   We thank the reviewer for their time taken to review our paper, and for their valuable suggestions that improve the quality, clarity and contribution of the manuscript.

   General comments:

2. *My main comment is that, because of the way the paper is structured, with the Methods at the end, it is rather hard to understand the design and logic of the experiment. For instance, when you were discussing spatial variation in the introduction, I assumed that you were talking about the informational content of spatial cues, as this is central to what immigrants need to learn about. For instance, an immigrant may need to learn something like "in this new environment, the best caterpillars are found on oak trees". Figure 1 also gave me this impression, as it seemed from the pictures that the spatial location of cues (deciduous or pine) was associated with the reward distribution in a meaningful way (see specific comment below). It was only much later that I realised that when you referred to spatial variation you meant habitat differences - i.e. moving from one habitat type to another, but with no new informative spatial cues about where food might be found.*

   We apologize it was not made more clear what we intended by the use of spatial variability. We intend spatial variability in a very broad sense, that not only habitat cues could be variable across a transect (observable variability), but also reward structures of familiar behaviors (un-observable variability). Related to your example, it might not be obvious that the best caterpillars are in oak trees, and learning this would require IL and/or SL. We have revised the first paragraph to set this up in a more explicit way, and telegraph our manipulation in the experiment:

   > "When animals move from one place to another through dispersal, migration or nomadism, they often experience spatial variability, whereby environmental factors change across a transect. Spatial variability can be observable (e.g. a change in habitat cues), but also might be un-observable, such as subtle changes in the rewards earned from familiar behavior. Exposure to observable variability may cause neurological changes that trigger learning [1], leading animals to explore the new environment to identify novel resources or risks [2, 3]. Yet, trial and error exploration and learning in new environments may be extremely costly. For example, toxicity risks of novel food items may be largely unknown to the individual [4]. Longstanding theory has thus predicted that it should be advantageous to preferentially rely on social information as a less costly alternative to individual learning [5, 6]..."

3. *Similarly, I did not immediately see the link between temporal variation and changes in payoffs - after all, if I am used to getting food rewards from the right-hand side but now the best rewards are on the left, that is a spatial, as well as a temporal change. I think providing a bit more detail on how the concepts covered in the introduction relate to the experimental design, and on how the*

experiment itself is structured, would really help readers to understand the study and reduce the need to flick back and forth between sections to make sense of it all.

We apologize for the lack of clarity in our description of how spatial/temporal variability map onto our design, and also note that this comment echoes comment 4 of Reviewer 3. We agree that any spatial variation is also experienced as temporal variation from an individual's point of view. Purely temporal variation in payoffs, where payoffs of all puzzle boxes were suddenly switched irrespective of physical location, was already tested in an experiment conducted in the wild by Aplin et al 2017. To resolve this concern and improve the clarity of our design choice, we now are careful to frame the current study in the context of this previous experiment, since it did not test spatial variability:

> "Prior empirical studies have shown that naive great tits can acquire foraging behaviors with a conformist transmission bias, meaning that they tended to learn the most frequently observed option [7, 8, 9, 10]. A follow up study found that this conformist bias did not create a trap in temporally variable environments where the rewards of options abruptly changed [8]. Tits adjusted their behavior to this change by relying mostly on individual learning, but retained a frequency-dependent conformity production bias [8]. However, the effect of spatial variability on tits' social learning has not yet been tested, even though spatial variability is hypothesized to drive the evolution of social learning strategies moreso than temporal variability [11], and could be more likely to trigger adaptive changes in learning strategies."

(The rationale of why spatial should be more important than temporal is now clarified in the preceding paragraph of the manuscript, see specific comment 10 below).

We now avoid referring to the P_a condition as temporal variability, and instead frame it as spatial variability throughout the manuscript. However, we do mention that it could be compared to the purely temporal variation found in Aplin et al. 2017.

To more clearly communicate the experimental design we have remade Figure 1 to have 3 more explicit panels that describe the immigration, puzzle boxes and hypothesis grid. We now specifically note that the seeded preferences of tutors were balanced across traditions both in the caption of Figure 1, and in the main text introduction:

> "We note that seeded traditions were balanced across the design to avoid any confound with a bias for a particular solution (which we did not find)."

4. *There were also aspects of the results that I found hard to follow. In particular, at first I found it difficult to reconcile Fig 2, where immigrants in the Symmetric Environment, Asymmetric Payoff (Es, Pa) show a clear switch to the resident strategy, with later arguments about limited learning from residents when environments were symmetrical. The discussion later clarifies that this is because Es; Pa immigrants relied more on individual learning about the optimal solution, but this was not immediately obvious from the presentation of Results. Perhaps you could include a figure to illustrate how reliance on individual learning varies across conditions?*

We apologize and agree that this difference in learning mechanism, as shown by the reinforcement models, should be clearly highlighted early on in the results to avoid any confusion. As part of the revision, we have now flagged this early on in the results:

> "Over the entire 14 day period following immigration, immigrants in the asymmetric environment and payoff condition produced a larger proportion of resident solutions (proportion per condition compared to the other conditions: $E_a, P_a = .849$, $E_s, P_a = .676$,

$E_a, P_s = .299$, $E_s, P_s = 0.014$)... Immigrants in the E_s, P_a condition also largely adopted the resident solution, but with a delay relative to the E_a, P_a condition (Figure 2, Figure 3). This delay hinted at a difference in learning mechanism, which we return to in the following selection."

Specific comments:

5. *Abstract and L82: State explicitly that experiments were in captivity. Given the authors' previous work, readers may assume (as I did) that the experiments were in the wild.*

   We have now explicitly stated this in the abstract and main text:

   > "We asked whether migration between spatially variable environments affected social information use with a large-scale cultural diffusion experiment with captive wild great tits (*Parus major*), a small passerine bird that can socially learn novel behaviors."
   >
   > "In order to test this hypothesis, we conducted a cultural diffusion experiment in captivity using 18 micro-populations..."

6. *L56: It would be useful to clarify here why resource scarcity should generate costs of social information acquisition*

   We have now included some examples of increased costs due to resource scarcity:

   > "Furthermore, the social learning might also come at some cost, especially under resource scarcity, either as an opportunity cost for time spent observing rather than foraging, or as increased likelihood of aggressive interactions due to resource defense [12]."

7. *L58: Typo - "should not copying"*

   We have amended this sentence.

8. *L64-71: There are some relevant studies beyond primates that could be cited here - e.g. migratory flycatchers learning from resident great tits in Finland; immigrant meerkats adopting the waking-up times of their new groups.*

   We thank the reviewer for these very relevant suggestions and have now included them:

   > "There have been several key studies which give insight into how immigrants use social information. Migrant pied flycatchers (*Ficedula hypoleuca*) were shown to use and benefit from social information gleaned from resident great tits in the context of nest site selection [13, 14]. Newly immigrated meerkats (*Suricata suricatta*) were found to adopt the wake-up time of residents of their new social group [15]. In chimpanzees..."

9. *L73: I suggest defining conformist transmission bias here for readers who may not be familiar with the formal definition.*

   We have now included a brief description:

   > "...great tits can acquire novel foraging techniques with a conformist transmission bias, meaning that they they disproportionately learned the most frequently observed option..."

10. *L76: Why is spatial variation thought to be more important than temporal variation? A brief explanation would be helpful for context.*

    We have now provided a paragraph that better sets up the rationale behind the differences in variation:

> "Formal models and experiments have addressed why immigrants should strategically use social learning after moving to a new environment. Firstly, under spatial variability, migration can introduce non-adaptive behavior that favors strategic learning that acts as a filter for both immigrants and residents [11]. This filtering is not as useful under temporal variability, as the entire population might possess non-adaptive behavior after a temporal change. Furthermore, migration results in experience-structured populations where residents would be preferred demonstrators because they might have gained more adaptive information as a result of longer experience in a particular environment [16]. A recent virtual foraging experiment on humans supported this difference between types of variability, showing that immigration across spatially variable environments temporarily increased reliance on social information more-so than temporal changes in rewards [16]."

11. *Figure 1: I found the figure a little hard to follow. In A, the pictures seem to indicate that environmental cues were spatially associated with one option on the puzzle box (e.g. deciduous foliage associated with the right-hand side, buffalo worm reward on the top left panel). It was only later that I realised this was not the case. It is also not clear what the blue font means. It would be helpful if the figure could clearly indicate which is the optimal (highest value) choice for immigrants.*

    We agree that this figure does summarize the design of the experiment very well. We have remade the figure to be more explicit and comprehensive. We have also noted in the caption that the side choices were balanced within each condition, and the sides shown in the figure are exemplary.

12. *In panel B, I wasn't sure why there were two symbols (+ or -) in each quadrant. E.g. if each quadrant represents a prediction about whether immigrants would adopt the resident solution, what does +- mean? Are the colours significant here (e.g. do they relate to the red and blue of the deciduous/pine foliage, or to door colour?).*

    We apologize for the lack of clarity in this panel. The +/- signs were intended to indicate the effect of either condition on the probability of adopting the resident solution, e.g. asymmetric conditions increased the chance, while symmetric conditions decreased the chance. We have replaced this panel with a more explicit description of our hypotheses.

13. *L130: It's interesting that the immigrants seemed to settle in immediately with no stress and were not subject to aggression by residents. To put the experiment into context, it would be useful if the introduction could tell us a bit more about immigration events in the wild. Do immigrant birds immediately start joining in social foraging events, and are they left in peace, as in the experiment? This has important implications for the potential to observe and learn from residents.*

    We apologize for this oversight, and realize that we did not provide enough context about great tits social life during the winter. We now provide relevant details in the introduction:

    > "Great tits' social system and learning abilities have made them a proven study system for questions linking social learning to social network structure and dynamics. During the winter months, great tits adopt a fission-fusion social system whereby birds join and leave mixed-species foraging flocks over the course of the day [17]. Tits are more socially tolerant compared with the spring breeding season, and belonging to winter flocks enhances their foraging abilities, either for patch discovery or the social transmission of novel foraging behavior [18, 7, 19, 20]..."

14. *L155: "although this did not meet our criterion for significance" - what is the criterion for significance?*

    We have amended this line to include this information:

"... although this did not meet our criterion for significance of $p < 0.05$..."

15. *L182: "When immigrants were socially influenced in the Es; Pa condition, they were estimated to be more influenced by observations of the lower payoff solution by fellow immigrants." If immigrants were more influenced by other immigrants, then why do they still appear to switch to the resident preference in Fig 2? Greater clarity on the role of individual learning would help here.*

    We apologize for this lack of clarity, and have made explicit here what was implied: that the switch over was due to individual learning.

    > "When immigrants were socially influenced in the E_s, P_a condition, they were estimated to be more influenced by observations of the lower payoff solution by fellow immigrants (Figure 4B). Their eventual shift towards the residents' preference was thus more-so driven by individual learning, rather than social influence from residents."

16. *L192: Similarly, the statement that "when environments were symmetric, immigrants... were more likely to ignore higher payoff solutions produced by residents" initially seems at odds with Fig 2. In the figure, it seems that in the Es, Pa condition immigrants still ended up choosing the resident solution around 60% of the time (Es; Pa = 0.676 on line 143). I can see that they are "more likely" to ignore the resident solution relative to the Ea, Pa condition (although the difference isn't huge) but overall it seems they are still pretty keen to switch to the resident solution when the environments are symmetrical. A clear explanation of how this comes about (through individually learning the optimal solution?) would be helpful.*

    Thank you, this suggestion will also improve the clarity of the results. The reviewer is correct, while they come to use the resident solution, they do so with a delay compared to EaPa (taking between 3-6 days to reach a comparable average preference level to E_a, P_a (Figure 3), and the model results suggest that this quick switch in E_a, P_a is attributable to social information use, while E_s, P_a immigrants changed preferences due to IL, and perhaps were further delayed in switching due to social information from fellow immigrants. We have now made this more explicit in the results.

    > "When taken together with the general pattern in Figure 2, these mechanisms point to a clear behavioral difference between the E_a, P_a and E_s, P_a conditions: immigrants rapidly changed their preferences using social information when experiencing asymmetric environments, and were disproportionately influenced by the social observation of asymmetric payoffs. Meanwhile, when environments were symmetric, immigrants were less influenced by social information, and they did not disproportionately attend to the higher payoff solutions. Individual learning was the most likely mechanism behind their adoption of the resident preference."

17. *Results - general query. Given the authors' previous work on conformist social learning, I was wondering throughout whether and how the relative numbers of resident solvers and immigrants matter. In some groups only 3 non-tutor residents learned to solve the task, whereas in others all 7 non-tutors were solvers. As immigrants will also see the attempts of fellow immigrants (plus a small number of non-tutor solutions by residents), the proportion of resident tutor solutions they are exposed to may be rather variable. Do the models account for this, and if so to what degree are immigrants affected by (a) the absolute number of times they witness the resident tutor solution, (b) the number of individuals using the resident tutor solution or (c) the proportion of solves that use the resident tutor solution vs the other option?*

    This is a very good question. We did try fitting several variations of conformist strategy models and could not get the models to converge. With regards to the question about

tutors, we had not tried to fit a model that treated tutor residents separately from non-tutors (only new associates vs old associates). As part of the revision we fit a model that allows for this distinction with a beta coefficient that controls the influence of tutor solves vs. non-tutor solves. We found no evidence that immigrants were disproportionately influenced by the observation of tutors versus non-tutors, and have briefly mentioned this in the results with parameter estimates reported in Figure S6:

> "Finally, we fit an ancillary model that estimated a tutor bias, but found no evidence for the disproportionate influence of tutors on immigrants (Figure S6)."

18. *L212: Typo - "can be exhibit"*

    We have amended this sentence.

19. *L226: "a payoff bias is less costly" than what?*

    We apologize for the lack of clarity. This paragraph now reads:

    > "We would suggest that a payoff bias is less costly than a conformist strategy for great tits, especially given their winter fission-fusion social system. Firstly, it may be difficult for immigrants to assess which of their group members are residents, as opposed to other immigrants from a third location. Secondly, the number of residents may be less than the number of immigrants, leading to a case where the frequencies of behaviors are not indicative of their adaptive value. This was also the case in our experiment, where residents varied substantially in their solving rate, leading to some replicates where the large majority of behavioral information was only available from one resident. Thirdly, we argue that payoff assessment is a cognitively straight-forward task in our design, since immigrant birds are already familiar with both buffalo worms and mealworms, and these food items are visually distinct with mealworms being about twice as large as buffalo worms. Birds carry the food items away from the puzzle in their bill before processing and eating them. Immigrants only had to attend to others' solutions and recognize the reward earned by the choice of a conspecific was a food item that they preferred more-so than the reward they had been earning from their own behavior. In open fission-fusion systems, the observation of payoff cues is therefore likely a more reliable means of identifying adaptive behavior. Thus, this is a case where theory and natural ecology of a species match to experiment."

20. *L249-252: It would be useful to present some of this information on natural patterns of migration in the Introduction, to give context to the study. For example, until I got to this point, I was wondering whether it is really realistic to think that great tits migrate between totally different habitat types.*

    We completely agree, and this echoes the point from comment 13. We now have included more context about the natural behavior of tits in the introduction as a part of the response to comment 13.

Reviewer 2:

1. *The current study reports a social learning experiment in wild-caught great tits, examining how the social and physical environment influences social learning in immigrants. After learning a puzzle box solving behaviour, birds were experimentally relocated into a population with residents which had learned an alternative solution. This new population was housed in either a symmetric or asymmetric physical environment, and the puzzle box solution either produced an identical or higher-payoff*

reward. Immigrants learnt more rapidly when both the physical environment and payoff changed. The study is a valuable contribution to the literature, particularly as it teases apart the influence of social and physical environment on social learning in immigrants, something which is highly challenging in wild populations. This type of experimental study in taxa in which experimental relocation is feasible and ethically acceptable is of great value in informing more observational work in primates or large mammals, with which such studies would be impossible to achieve. I therefore think it will be of great interest to a broad range of researchers. Overall I found the study design very thorough and well-focused on the authors' questions. The manuscript is very well written and cites the appropriate literature. I therefore have only very minor comments.

We thank the reviewer for their time spent reviewing our manuscript and their encouraging comments.

Minor comments:

2. *Typo in line 58 "social learning individuals should not copying all information"*

   We have amended this sentence.

3. *Line 70: "male immigrants employed a pay-off biased learning strategy that overrode their model bias, while philopatric females continued to prefer to learn from other dominant females" I believe that in this study both juvenile and adult males were tested, so results from both resident and immigrant males were combined when finding the sex effect referred to here. I think the authors' point stands but to my knowledge the cited study did not explore whether immigrant adult males differed from resident juveniles in their learning preference (i.e. the found result suggests a payoff bias in the dispersing sex, but not necessarily an effect of migration) so this should perhaps be rephrased. This comes up again in line 217 - it may well be that the result found in the original study was driven by immigrant males, but to my knowledge it was not analysed in a way that would allow this conclusion to be drawn as adult and juvenile male data were combined.*

   We thank the reviewer for this helpful note, we had missed this subtlety about the study. We have now amended these sentences in the introduction to avoid directly stating that they were actually immigrants, but rather the dispersing sex:

   > "In vervet monkeys (*Chlorocebus pygerythrus*), males (the dispersing sex) were shown to preferentially copy dominant females who form the stable core of their social system [21]. However, in this case, when rewards for behaviors were varied, males employed a pay-off biased learning strategy that overrode their model bias, while philopatric females continued to prefer to learn from other dominant females [22]."

   > "Specific reliance on a pay-off biased strategies by immigrants has been demonstrated in captive chimpanzees [23], and in male vervet monkeys, which are the dispersing sex [22]."

4. *Line 262-263: "we further show that these changes in learning strategies are not due to underlying life-history state, e.g. tied to hormonal changes during dispersal". This is certainly the case within the current experiment, but I'm not convinced this conclusion can be generalised from the current study to rule out this causal mechanism as a contributing factor in other species? I.e. the fact that these learning strategies can be elicited via experimental immigration events alone does not exclude the possibility that hormonal changes during dispersal contribute in other observations. Perhaps these would have an additive effect (an increased attention to social information, or more rapid learning, for example).*

   We thank the reviewer for this insightful comment and have clarified our position. We agree that both mechanisms could operate in different contexts/species

"By inducing immigration events in controlled conditions, we further show that these changes in learning strategies, at least in great tits, are not due to underlying life-history state, e.g. tied to hormonal changes during dispersal [24]. Rather, they represent flexible shifts triggered by experience of new environments. However, we do not discount the role that hormonal changes may play in other contexts or species, and suggest that both mechanisms might operate in an additive effect."

5. *Figure 2 is a very clear communication of the results (I received the updated version prior to review). The statistical analysis appears appropriate and thorough.*

   We again thank the reviewer for their helpful comments that have improved the quality and clarity of our manuscript.

Reviewer 3:

1. *The value and use of social information is an issue that is highly topical with a broad reach in terms of audience who should be interested in the data presented here. I think the experiment is clearly explained, conducted well, and the data analysed appropriately. I do, however, have a question or two regarding the rationale, the appropriateness of the experimental design for this question, and the interpretation of the data, which leave me thinking that the authors are missing some key parts to this story.*

   We thank the reviewer for their careful comments, and hope that our revisions in response have improved the clarity and value of the manuscript.

2. *L40: nothing further is said here about the resident responses to immigrants and there is only a very little further in the text. This appears to be a strong and rapid result, albeit not long-lived but I do not see any explanation. I am not sure how the data shown in Figure S4 map to the data in Fig 2?*

   While the original focus of our study was on immigrant behavior, we completely agree with the reviewer that the perturbation in resident preferences is an important aspect of the results. In response to this comment and comment 4 below we have put more emphasis on this interesting result. We have now discussed the aspect of social variability in the introduction, included a hypothesis regarding the residents, and added a paragraph related to residents in the Results section. We have also included a supplementary figure that further details resident preferences after immigration. Please see our response to comment 4 below for the specific changes we have made.

   Related to figure S4, we have specifically addressed how Figure S4 (now S5) relates to Figure 2 in the main text:

   "We found evidence that residents in both symmetric payoff conditions were influenced by immigrant solutions (residents in E_a, P_s: $\hat{\beta}_n = 1.03(-0.03, 1.95)$, E_s, P_s: $\hat{\beta}_n = 0.93(-0.67, 2.45)$), whereas there was no evidence that residents were influenced by immigrant solutions in asymmetric payoff conditions, suggesting that residents were assessing the rewards and were not influenced by the lower-rewarded option (Figure S5). This bias explained the perturbation in residents preferences following immigration in Figure 2."

3. *I missed a very clear hypothesis. There is a prediction that the impact of spatial variation should be greater than that of temporal variation on the probability of learning from others but I am not sure that the authors have clearly explained why this would be the case?*

We apologize for this lack of a clear explanation. This sentiment is also echoed by Reviewer 1 comment 10, who wished for more of an explanation as to why the theoretical models predict that spatial variation should be a stronger driver of learning strategies. We have added the following paragraph to the introduction that explains the rationale behind why spatial variability is an important driver of strategic learning:

> "Formal models and experiments have addressed why immigrants should strategically use social learning after moving to a new environment. Firstly, under spatial variability, migration can introduce non-adaptive behavior that favors strategic learning that acts as a filter for both immigrants and residents [11]. This filtering is not as useful under temporal variability, as the entire population might possess non-adaptive behavior after a temporal change. Furthermore, migration results in experience-structured populations where residents would be preferred demonstrators because they might have gained more adaptive information as a result of longer experience in a particular environment [16]. A recent virtual foraging experiment on humans supported this difference between types of variability, showing that immigration across spatially variable environments temporarily increased reliance on social information more-so than temporal changes in rewards [16]."

4. *Furthermore, in the experiment that has been conducted there is clear spatial variation (here a change in some of the physical aspects of the environment), which then sets the scene for attention to change in food reward/the behaviour of the other individuals. I think the other cue that has changed is the identity of the individuals to which the immigrants are exposed (as the authors also finally suggest in lines 252-253). It appears that by being moved to a different environment (the asymmetric environment), this change in physical appearance is big enough that the birds have been cued to look for difference. They then immediately pay attention to the residents, learning rapidly (stability within some 10 trials) in both instances to switch or stick with their response to the puzzle (ie the response learned in the previous environment). The move to the 'same'/symmetric environment does not, however, alert the birds with the asymmetric payoff in the same way so they pay less attention to the residents. Over time, however, they begin to do so, and then begin to switch their response. This doesn't seem to me to be a test of spatial versus temporal information but rather of spatial versus social information (here identity of new group mates). Possibly because these birds do move around with a flexible number and identity of flock mates, this information is simply less salient to them. I must have missed something and may well be using the wrong language (e.g. words like attention) to describe the data. Paying attention to flock mates is sometimes also something the residents also do. The data show that when the residents are getting better food, they ignore the behaviour of the immigrants (upper 2 panels in Fig 2), but they pay immediate attention (and copy) the behaviour of the immigrants in the alternative payoff situation. The temporal component is that the spatial change occurs before the availability of information about food reward but isn't really testing the importance of one up against the other as, as I understand it, the authors seem to suggest they have done. Clarifying this would be hugely helpful.*

   We apologize for this general confusion over spatial and temporal variation, which was likely caused by the ambiguous start of the paragraph on L82: "In order to test this hypothesis..." following the description of Deffner at al, which did test temporal and spatial variation, and then reinforced by the first sentence of the discussion, which read "By simulating immigration events for individuals between groups with different traditions in different environments (simulating spatially variable environments) or different foraging pay-offs (simulating temporally-variable environments) we could disentangle flexibility in social learning strategies associated with these two sources of variation."

   Firstly, we recognize that we did not test pure temporal variability, which would have been leaving the birds in the same aviary but manipulating the payoffs (similar to the design

of Deffner et al 2021 or Aplin et al 2017). Rather we linked the change in payoffs in the P_a condition to a change in physical location, making it more like spatial variation in resources/rewards. In our revision, we have rewritten the introduction to emphasize only the focus on spatial variation in the current design, rather than framing it as if we've tested spatial versus temporal. We have modified the introduction to clarify this:

> "... Tits adapted their behavior to this change by relying mostly on individual learning, but retained a frequency-dependent conformity production bias [8]. However, the effect of spatial variability on tits' social learning has not yet been tested, even though spatial variability is hypothesized to drive the evolution of social learning strategies more-so than temporal variability [11], and could be more likely to trigger adaptive changes in learning strategies.
>
> To test the effect of spatial variability on social information use, we conducted a cultural diffusion experiment in captivity ..."

We also modified the beginning of the discussion to remove reference to temporal variation, and emphasize the results regarding social variability:

> "By simulating immigration events for individuals between groups with different traditions in different environments or different foraging pay-offs, we could disentangle flexibility in learning strategies associated with these two sources of variability. Our design also allowed us to test birds' responses to social variability alone."

Secondly, the reviewer is exactly correct in all 4 conditions, both immigrants and residents changed social partners. For residents specifically, social partners changed without any change in their environment, even any minor changes that might have been noticed in the E_s conditions by immigrants. We now are careful to introduce this point from the outset, telegraphing this to the reader early in the introduction:

> "One particularly uncertain context for wild animals is after migration or dispersal, as they are exposed to new physical and social environments. Several key studies give insight into how immigrants use social information..."

And now explicitly mentioned in the setup of the methods at the end of the introduction:

> "Finally, our design meant that immigrants would change both physical and social environments, while residents would only change social environments. While this study is largely focused on the behavior of immigrants, residents provided valuable insight into behavioral changes caused by a change in social environment alone."

We provide a hypothesis for residents at the end of the introduction:

> "If social variability alone triggered social learning, we expected residents to be more likely to be influenced by immigrants in P_s conditions, since the mealworm reward would be more appealing that the buffalo worm reward obtained from the immigrant preference in P_a conditions."

We also now discuss residents' behavior early in the results in the following added paragraph, and have added a supplementary figure S2 to illustrate the residents preferences over the entire course of the experiment:

"We also found interesting perturbations in the preferences of immigrants and residents in the P_s conditions where both immigrants and residents appeared to be influenced by each other in the short term. Two immigrants came to prefer the resident preference (Figure 3 E_a, P_s condition), and surprisingly, nine residents used the immigrant preference more than 50% of the time (Figure S2 P_s conditions). Two of these had learned the puzzle after immigration, potentially socially learning from observations of immigrants. The commonality between the remaining seven were that they were very low-frequency solvers (range: 6-89 total solves) compared to the median (1128 total solves). We infer from this pattern that social variability can increase reliance on social information, as birds generally did not sample the opposing option prior to immigration. However, social influence was not generally strong enough to overcome the value of their original preference, gained through previous experience."

5. *I think it would also help put the interpretation of the data into a broader context that was about the mechanistic basis of learning and not just how information might be put to use once an animal has acquired it. Here the authors explain their data in the context of high uncertainty but it is not clear how they have tested high temporal uncertainty.*

   We thank the reviewer for the suggestion to add more detail about the mechanistic basis of learning (which we interpreted as cognitive and physiological bases). If we have omitted any citations or critical discussion points here, we would appreciate it if the reviewer pointed them out to us in their response. As part of the revision we have included the following paragraph:

   "The results of our experiment demonstrate that, in tits, social learning is not inflexible or unbiased, but rather can exhibit adaptive flexibility in response to environmental change or uncertainty [5, 25, 26, 27, 28, 29, 30]. Indeed, for over a century it has been recognized that phenotypic plasticity, or the expression of different phenotypes depending on environment, is crucial for success in adapting to novel environments [31, 32]. Our current study points to specific plasticity in learning strategy to changes in the environment. Our learning model analysis indicates the cognitive mechanism responsible for immigrants' rapid adoption of resident preferences in the E_a, P_a condition was the activation of a learning strategy where the rewards of others were accounted for when making future decisions. By inducing immigration events in controlled conditions, we further show that these changes in learning strategies, at least in great tits, are not due to underlying life-history state, e.g. tied to hormonal changes during dispersal [24]. Rather, they represent flexible shifts triggered by experience of new environments. However, we do not discount the role that hormonal changes may play in other contexts or species, and suggest that both mechanisms might operate in an additive effect."

   As for the second point, again we apologize for the ambiguity in the first submission over temporal variation. We hope that our revisions in response to the prior comment have made it clear that we are explicitly testing spatial variation in environmental cues, rewards for the options, and social partners.

   Details:

6. *L219: is there spatial variability in the payoff cues? Any idea why the immigrants in the asymmetric payoff conditions never get above 75%?*

   Regarding the first question, we would argue that there is spatial variability of payoffs in the sense that the reward structure of the puzzlebox had changed in their new environment.

For immigrants, the alternative side changed from providing low-value sunflower seeds to providing high-value mealworms. We have now clarified this sentence:

> "The results of our experiment add to these findings, yet suggest that tits do not always adopt the preferences of their new group, but rather that this depends both on whether there is spatial variability in payoffs (in the sense that rewards of options has changed with physical location) and environmental cues."

Regarding the second question, the data shown in Figure 2 is limited to the first 200 solutions produced after immediately after the immigration event (which is the data used to fit the learning models). Figure 3 shows the entire time-series in the 14 days, and in both the asymmetric payoff conditions birds converge closer to 100% at around day 6. We used the first 200 solutions in the learning model because our interest was in explaining mechanism underlying the differing pattern of adoption, where E_a, P_a immediately began using the resident solution, and E_s, P_a had a slower adoption curve.

7. *Overall, I think the writing could do with some editing to make it more accessible to the audiences I think the authors might like to reach. Currently there is a serious expectation that the readership is very familiar with the language and background: from assuming that Rogers' (I think the apostrophe in the text might be in the wrong place?) paradox is known outside the social learning crowd to the discussion in lines 240-244. If the writers give the readers less work to do they will feel more clever, and be more impressed by the data.*

   We completely agree that we should have provided more context to make the paper more accessible to readers who might not come from the SL field. We have rewritten the introduction to be more accessible with attention to the first paragraph:

   > "... Longstanding theory has thus predicted that it should be advantageous to preferentially rely on social information as a less costly alternative to individual learning [5, 6]. However, as the proportion of social learners increases, they rely on an ever smaller set of individual learners who are actively sampling the environment, causing the average fitness of the population to fall—an outcome referred to as Rogers' paradox [33]. Furthermore, the social learning might also come at some cost, especially under resource scarcity, either as an opportunity cost for time spent observing rather than foraging, or as increased likelihood of aggressive interactions due to resource defense [12]."

References

1. Park AJ, Harris AZ, Martyniuk KM, Chang CY, Abbas AI, Lowes DC, Kellendonk C, Gogos JA, and Gordon JA. Reset of hippocampal–prefrontal circuitry facilitates learning. Nature 2021; 591:615–9
2. Mettke-Hofmann C, Rowe K, Hayden T, and Canoine V. Effects of experience and object complexity on exploration in garden warblers (Sylvia borin). Journal of Zoology 2006; 268:405–13
3. Dingemanse NJ, Both C, Drent PJ, and Tinbergen JM. Fitness consequences of avian personalities in a fluctuating environment. Proceedings of the Royal Society of London. Series B: Biological Sciences 2004; 271:847–52
4. Galef Jr BG, Dudley KE, and Whiskin EE. Social learning of food preferences in 'dissatisfied'and 'uncertain'Norway rats. Animal Behaviour 2008; 75:631–7
5. Boyd R and Richerson PJ. Culture and the evolutionary process. University of Chicago press, 1988
6. Aoki K and Feldman MW. Evolution of learning strategies in temporally and spatially variable environments: a review of theory. Theoretical population biology 2014; 91:3–19
7. Aplin LM, Farine DR, Morand-Ferron J, Cockburn A, Thornton A, and Sheldon BC. Experimentally induced innovations lead to persistent culture via conformity in wild birds. Nature 2015; 518:538
8. Aplin LM, Sheldon BC, and McElreath R. Conformity does not perpetuate suboptimal traditions in a wild population of songbirds. Proceedings of the National Academy of Sciences 2017; 114:7830–7
9. Chimento M, Alarcón-Nieto G, and Aplin LM. Population turnover facilitates cultural selection for efficiency in birds. Current Biology 2021; 31:2477–83
10. Wild S, Chimento M, McMahon K, Farine DR, Sheldon BC, and Aplin LM. Complex foraging behaviours in wild birds emerge from social learning and recombination of components. Philosophical Transactions of the Royal Society B 2022; 377:20200307
11. Nakahashi W, Wakano JY, and Henrich J. Adaptive social learning strategies in temporally and spatially varying environments. Human Nature 2012; 23:386–418
12. Gruber T, Chimento M, Aplin L, and Biro D. Efficiency fosters cumulative culture across species. Philosophical Transactions of the Royal Society B 2022; 377:20200308
13. Moenkkoenen M and Forsman JT. Heterospecific attraction among forest birds: a review. Ornithological science 2002; 1:41–51
14. Forsman JT, Thomson RL, and Seppänen JT. Mechanisms and fitness effects of interspecific information use between migrant and resident birds. Behavioral Ecology 2007; 18:888–94
15. Thornton A, Samson J, and Clutton-Brock T. Multi-generational persistence of traditions in neighbouring meerkat groups. Proceedings of the Royal Society B: Biological Sciences 2010; 277:3623–9
16. Deffner D, Kleinow V, and McElreath R. Dynamic social learning in temporally and spatially variable environments. Royal Society Open Science 2020; 7:200734
17. Perrins CM. British tits. Vol. 62. HarperCollins, 1979
18. Aplin LM, Farine DR, Morand-Ferron J, and Sheldon BC. Social networks predict patch discovery in a wild population of songbirds. Proceedings of the Royal Society B: Biological Sciences 2012; 279:4199–205
19. Farine DR, Aplin LM, Sheldon BC, and Hoppitt W. Interspecific social networks promote information transmission in wild songbirds. Proceedings of the Royal Society B: Biological Sciences 2015; 282:20142804

20. Beck KB, Sheldon BC, and Firth JA. Social learning mechanisms shape transmission pathways through replicate local social networks of wild birds. elife 2023; 12:e85703
21. Van de Waal E, Renevey N, Favre CM, and Bshary R. Selective attention to philopatric models causes directed social learning in wild vervet monkeys. Proceedings of the Royal Society B: Biological Sciences 2010; 277:2105–11
22. Bono AE, Whiten A, Schaik C van, Krützen M, Eichenberger F, Schnider A, and Waal E van de. Payoff-and sex-biased social learning interact in a wild primate population. Current Biology 2018; 28:2800–5
23. Vale GL, Flynn EG, Lambeth SP, Schapiro SJ, and Kendal RL. Public information use in chimpanzees (Pan troglodytes) and children (Homo sapiens). Journal of Comparative Psychology 2014; 128:215
24. Mörchen J, Luhn F, Wassmer O, Kunz J, Kulik L, Van Noordwijk M, Van Schaik C, Rianti P, Atmoko SSU, et al. Migrant orangutan males use social learning to adapt to new habitat after dispersal. Frontiers Ecology And Evolution 2023; 11
25. Laland KN. Social learning strategies. Animal Learning & Behavior 2004; 32:4–14
26. McElreath R, Lubell M, Richerson PJ, Waring TM, Baum W, Edsten E, Efferson C, and Paciotti B. Applying evolutionary models to the laboratory study of social learning. Evolution and Human Behavior 2005; 26:483–508
27. Kendal RL, Coolen I, Bergen Y van, and Laland KN. Trade-offs in the adaptive use of social and asocial learning. Advances in the Study of Behavior 2005; 35:333–79
28. Kendal J, Giraldeau LA, and Laland K. The evolution of social learning rules: payoff-biased and frequency-dependent biased transmission. Journal of theoretical biology 2009; 260:210–9
29. Mesoudi A. An experimental comparison of human social learning strategies: payoff-biased social learning is adaptive but underused. Evolution and Human Behavior 2011; 32:334–42
30. Kendal RL, Boogert NJ, Rendell L, Laland KN, Webster M, and Jones PL. Social learning strategies: Bridge-building between fields. Trends in cognitive sciences 2018; 22:651–65
31. Baldwin JM. A new factor in evolution. American Naturalist 1896 :441–51, 536–53
32. Yeh PJ and Price TD. Adaptive phenotypic plasticity and the successful colonization of a novel environment. The American Naturalist 2004; 164:531–42
33. Rogers AR. Does biology constrain culture? American Anthropologist 1988; 90:819–31
